# Uptake rate of influenza vaccines and its associated factors among patients with asthma or chronic obstructive pulmonary disease in Klang Valley: A health belief model study

**DOI:** 10.51866/oa.758

**Published:** 2026-04-09

**Authors:** Ainul Basyirah Mohd Azizi, Ahmad Nazmi Zaini, Aidi Zaharudin, Bavani Poobalan, Choh Ken Yee, Seng Fah Tong, Maizatullifah Miskan

**Affiliations:** 1 Klinik Kesihatan AU2, Taman Sri Keramat, Kuala Lumpur, Wilayah Persekutuan Kuala Lumpur, Malaysia.; 2 Klinik Kesihatan Bandar Kota Tinggi, Jalan Kota Kecil, Bandar Kota Tinggi, Kota Tinggi, Johor, Malaysia.; 3 Klinik Kesihatan Kalumpang, Kerling, Selangor, Malaysia.; 4 Klinik Mediviron Cyberjaya, Shaftsbury Square 2350, Unit P1-16, Persiaran Multimedia, Cyberjaya, Selangor, Malaysia.; 5 Klinik Kesihatan Long Lama, Dewan Masyarakat Long Lama, Kampung/Pekan Long Lama, Baram, Sarawak, 98050 Telang Usan, Miri, Sarawak, Malaysia.; 6 Hospital Canselor Tuanku Muhriz UKM, Jalan Yaacob Latif, Bandar Tun Razak, Cheras, Wilayah Persekutuan Kuala Lumpur, Malaysia.; 7 Universiti Pertahanan Nasional Malaysia (UPNM), Kem Sungai Besi, Kuala Lumpur, Malaysia.

**Keywords:** Influenza vaccines, Health belief model, Chronic obstructive pulmonary disease, Asthma

## Abstract

**Introduction:**

Influenza is a significant cause of morbidity among patients with asthma and chronic obstructive pulmonary disease (COPD). In Malaysia, the Ministry of Health recently updated the national formulary to include influenza vaccination for senior citizens aged 60 years and above with at least one chronic illness, effective from 18 February 2025. However, the uptake rate of influenza vaccination among patients with asthma and COPD remains unclear. This study aimed to determine the association between sociodemographic factors, health belief model components and influenza vaccine uptake among patients with asthma or COPD in Klang Valley.

**Methods:**

A cross-sectional survey was conducted among 200 adults with asthma or COPD attending follow-up at five primary care clinics in Klang Valley. Sociodemographic data, vaccination history and vaccination beliefs were recorded using a questionnaire based on the health belief model. Data were examined using univariate and multivariate analyses.

**Results:**

A total of 200 patients participated. The influenza vaccine uptake rate was 24.0%. The employed patients were significantly more likely to be vaccinated (odds ratio [OR] =3.68; 95% confidence interval [CI] = 1.09–12.38; P=0.036). Stronger cues to action increased uptake (OR = 4.04; 95% CI = 1.94–8.40; P = 0.001), with healthcare provider recommendation being the main contributing factor. Higher perceived barriers reduced uptake (OR = 0.41; 95% CI = 0.18–0.93; P = 0.032), particularly concerns regarding vaccine side effects.

**Conclusion:**

Influenza vaccine uptake among high-risk patients in Malaysia is low. Employment, healthcare provider recommendations and perceived barriers strongly influence vaccination behaviour.

## Introduction

Influenza is a respiratory tract infection that causes diseases ranging from mild upper respiratory tract infection to severe pneumonia. Typically, the incidence is higher during winter; however, in Southeast Asian countries, it occurs throughout the year. Influenza is associated with hospitalisations among patients with asthma and chronic obstructive pulmonary disease (COPD). Each year, influenza kills approximately 250,000–500,000 people and causes millions of illnesses.^1^ The respiratory viral infection is a known cause of an acute exacerbation of asthma or COPD, which increases the risk of morbidity and mortality.^2^ Evidence suggests that influenza vaccination is safe for patients with COPD and asthma.^3^ Guidelines recommend influenza vaccination to control symptoms and minimise future risks of exacerbation and hospitalisation. However, the rates of compliance with annual influenza vaccination have been low among these patients.

Data from developed countries such as the United Kingdom (UK) revealed an influenza vaccine uptake rate of 41.4% in 2024 among individuals aged 6 months to under 65 years who were at risk.^4^ Several studies have identified predictors of adherence to influenza vaccination in adult populations with asthma.^3,5^ The most consistently reported factors or predictors are healthcare providers recommendations,fear of adverse effects of the influenza vaccine and the peeceived ability of vaccination to protect from influenza infection.^6^ Health belief models (HBM) components consist of perceived susceptibility, perceived severity, perceived benefit, perceived barriers and cues to action. The reported predictors mentioned above correspond with cues to action, perceived barriers and perceived benefits.

However, in the Malaysian healthcare system, where a large proportion of patients with asthma or COPD are being followed up in government health clinics or hospitals, the influenza vaccine is not often promoted.Thie is because resources are limited to government settings and are targeted and offered to individuals aged 60 years and above with at least one chronic illness.

**Figure 1:**
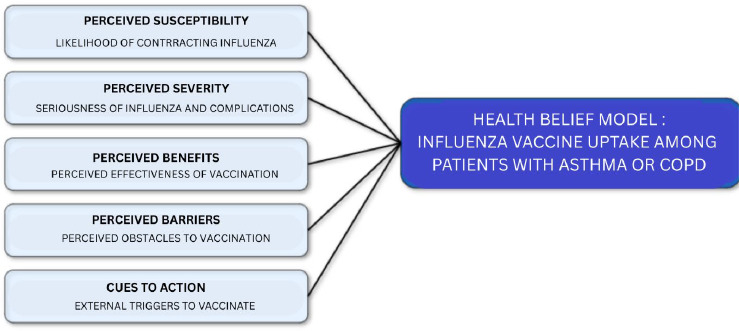
Conceptual framework of the HBM

Health behaviour car be examined in the context of individual ideas about health issues using the HBM. Figure 1 illustrates the conceptual framework of the camponents of the HBM concerning influenza vaccination uptake among patients with asthma and COPD.

The framework suggests that the chance of receiving the influenza vaccine will interact with the factors mentioned above. The framework thus presents a scenario where an individual’s belief and external cues can intervene in the target audience’s decision-making process – with this particular focus directed at patients with asthma and COPD.

The purpose of this research was to study the uptake of the influenza vaccine among patients with asthma and COPD and its association with sociodemographic factors and health beliefs about influenza vaccination. To achieve this, we conducted a cross-sectional study to assess the awareness of influenza vaccination uptake among patients with asthma and COPD.

## Methods

### Study design and data collection

This cross-sectional study was conducted among adult patients with asthma and COPD in five primary care clinics in Klang Valley from 20 May 2020 to 30 May 2021. All registered patients from five primary care clinics were invited to participate via opportunistic sampling. A total of 175 patients were from public primary care clinics, and 25 patients were from private clinics. These patients were recruited through appointments in asthma clinics and walk-in outpatient clinics. However, some patients did not accept the invitation; hence, the response rate was 83%. For this study, eligible participants included all patients who were 18 years old and above, were diagnosed with asthma or COPD, could read and speak English or Malay and had received a prescription for inhaled beta-2 (β2) agonists, antimuscarinic agents or inhaled/oral steroids in the past year.

Eligible participants were approached by the researchers and were informed regarding the study. They were also given patient information sheets. Patients who agreed to participate signed an informed consent form and proceeded with a self-administered questionnaire. The estimated duration for completing the questionnaire was 15–30 minutes. Once completed, the questionnaire was returned to the researchers. No token was given. This study was approved by the National Medical Research Register (NMRR) as well as the Medical Research Ethics Committee of Pusat Perubatan Universiti Malaya (PPUM).

The questionnaire was expanded and translated from multiple studies with the original author’s permission.^7^ Thereafter, it was reviewed by two family medicine specialists, and a face validity survey was conducted among 10 individuals with different backgrounds; their feedback and opinion regarding any confusion on the items, any medical jargon used and whether respondents had suggestions for possible improvements of the items were recorded. The questionnaire underwent a detailed validation process to ensure clarity and effectiveness.

**Figure 2:**
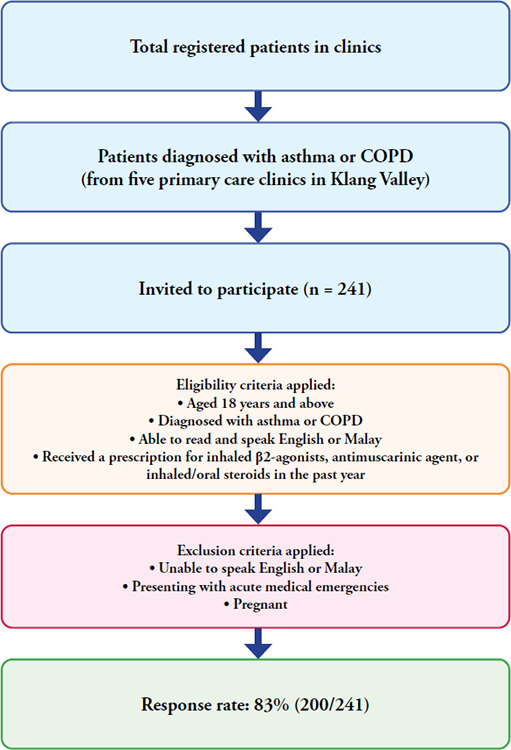
Flow diagram of participant recruitment and selection process, showing eligible participants (n = 241) and final response rate of 83% (200/241).

### Measurement tools

The questionnaire consisted of two parts. Part 1 included 11 questions about sociodemographic factors such as age, sex, marital status, race, educational level, average income, employment status and influenza vaccination status.

Part 2 comprised 16 statements based on the HBM, covering five domains: perceived susceptibility, perceived severity, perceived benefits, perceived barriers and cues to action. The statements are presented in Table 1. Each HBM statement on the influenza vaccine was scored on a 5-point Likert scale: strongly agree, agree, unsure, disagree and strongly disagree.

**Table 1:** Health belief model statements included in the questionnaire.

No.	Statement	HBM Domain
1	People with chronic illnesses such as asthma or COPD are at higher risk of developing severe influenza-related illness.	Perceived susceptibility
2	I am susceptible to influenza.	Perceived susceptibility
3	Catching influenza is not a major concern for me.	Perceived susceptibility
4	Avoiding crowded places helps prevent the spread of influenza.	Perceived susceptibility
5	Influenza is associated with serious complications.	Perceived severity
6	Influenza infection is more dangerous for patients with asthma or COPD than for others.	Perceived severity
7	Influenza infection can cause death.	Perceived severity
8	The influenza vaccine provides good protection against influenza.	Perceived benefit
9	Influenza vaccination can prevent acute exacerbation of asthma or COPD.	Perceived benefit
10	The influenza vaccine may cause harm to me.	Perceived barrier
11	The influenza vaccine can cause side effects.	Perceived barrier
12	The influenza vaccine can cause influenza.	Perceived barrier
13	My doctor or nurse advised me to receive the influenza vaccine.	Cue to action
14	My friends or family advised me to receive the influenza vaccine.	Cue to action
15	I would receive the influenza vaccine if it were offered free of charge by the government.	Cue to action

*HBM = Health Belief Model; COPD = chronic obstructive pulmonary disease.* *Domains reflect HBM constructs: perceived susceptibility, perceived severity, perceived benefit, perceived barrier, and cues to action).* *Item 3 is negatively worded statement; reverse coded during analysis.*

### Data analysis and sample size calculation

The sample size of200 patients was calculated using the OpenEpi Sample Size Calculator (OpenEpi, Version 3.01, Open Source Epidemiologic Statistics for Public Health, Atlanta, Georgia, USA). The calculation was based on an expected influenza vaccination rate of 15%, with a level of confidence of 95% (Z=1.96) and a margin of precision of approximately 5%. The vaccination rate of 15% was chosen as an estimate due to lower rates in the United States of America (USA) (41.7%) and because it is in line with Singapore’s rate (15.2%), considering its demographics. After the standard sample size formula for proportions was applied: n=(Z^2^ × p × (1–p))/d2, the calculated sample size was estimated at around 196. For ease of execution, the figure was increased to 200.

Data were analysed using the Statistical Package for the Social Sciences (SPSS) software, version 26.0 (IBM Corp., Armonk, New York, USA). Descriptive analyses were performed to examine the sociodemographic profile and prevalence of self-reported uptake of the influenza vaccine among patients with asthma or COPD.

Likert-scale responses were assigned mean scores and transformed into categorical variables *(agree, unsure* and *disagree)* before the chi-square test and pooled t-test were used to examine the association between patient’s baseline characteristics or vaccination beliefs and self-reported adherence to influenza vaccination. Associations of sociodemographic factors and health beliefs regarding influenza vaccination (based on the HBM components) with the uptake rate of influenza vaccination were assessed. The best response for each domain was analysed using a multivariate model. Variables were selected based on the distribution of patients’ results from the correlation and univariate analyses. P-values of less than 0.05 were considered statistically significant.

## Results

A total of 200 participants met the inclusion criteria. The overall influenza vaccine uptake rate was 24.0%. Among the vaccinated participants, 64.6% received the vaccine within the past year, while 35.4% received it more than 1 year earlier.

Table 2 shows the sociodemographic profile of the participants. The mean age was 46.0 ± 2.7 years (range = 18–86). The majority were women (71.0%), married (60.0%) and Malay (69.5%). Additionally, most participants completed either secondary (41.5%) or tertiary education (45.0%) and were from the lower 40% income group (B40) (71.0%). More than half were employed (59.0%). The majority of the participants had asthma (92.0%), while only 8.0% had chronic obstructive pulmonary disease (COPD).

**Table 2:** Sociodemographic characteristics and health belief model components of the participants and their association with influenza vaccine uptake (bivariate analysis).

Variable	Category	Total n (%)	Vaccinated n (%)	P-value
Age (years)	Mean ± SD	46.0 ± 2.7	43.9 ± 17.3	0.687
Sex	Female	142 (71.0)	37 (26.1)	0.287
	Male	58 (29.0)	11 (19.0)	
	Single	56 (28.0)	15 (26.8)	0.819
Marital status	Married	120 (60.0)	27 (22.5)	
	Others	24 (12.0)	6 (25.0)	
	Malay	139 (69.5)	33 (23.7)	0.820
Race	Chinese	18 (9.0)	3 (30.0)	
	Indian	43 (21.5)	9 (20.9)	
	Primary	27 (13.5)	3 (11.1)	0.002^*^
Educational level	Secondary	83 (41.5)	13 (15.7)	
	Tertiary	90 (45.0)	32 (35.6)	
	B40	142 (71.0)	25 (17.6)	0.001^*^
Income group	M40	44 (22.0)	17 (38.6)	
	T20	14 (7.0)	5 (55.6)	
Employment status	Unemployed	82 (41.0)	11 (13.4)	0.003^*^
	Employed	118 (59.0)	37 (31.4)	
Illness	Asthma	184 (92.0)	46 (25.0)	0.261
	COPD	16 (8.0)	2 (12.5)	
	Disagree	28 (14.0)	8 (28.6)	0.752
Perceived susceptibility	Unsure	61 (30.5)	13 (21.3)	
	Agree	111 (55.5)	27 (24.3)	
	Disagree	15 (7.5)	4 (26.7)	0.158
Perceived severity	Unsure	18 (9.0)	1 (5.6)	
	Agree	167 (83.5)	43 (25.7)	
Perceived benefits	Disagree	12 (6.0)	6 (50.0)	0.023^*^
	Agree	153 (76.5)	38 (24.8)	
	Disagree	93 (46.5)	30 (32.3)	0.013^*^
Perceived barriers	Unsure	77 (38.5)	10 (13.0)	
	Agree	30 (15.0)	8 (26.7)	
	Disagree	40 (20.0)	5 (12.5)	0.005^*^
Cues to action	Unsure	16 (8.0)	0 (0.0)	
	Agree	144 (72.0)	43 (29.9)	

* Values are presented as number (percentage) unless otherwise stated. SD = standard deviation; HBM = Health Belief Model; B40 = bottom 40% income group; M40 = middle 40% income group; T20 = top 20% income group. COPD = chronic obstructive pulmonary disease. Statistically significant at P < 0.05.

Table 2 presents the sociodemographic characteristics and health belief model components of the participants and their association with influenza vaccine uptake.

Higher educational level (P = 0.002), higher income group (P = 0.001), and employment status (P = 0.003) were significantly associated with vaccine uptake. Among the health belief model domains, perceived benefits (P = 0.023), perceived barriers (P = 0.013), and cues to action (P = 0.005) were significantly associated with influenza vaccination.

**Table 3:** Multivariate logistic regression model identifying independent predictors of influenza vaccine uptake (Multivariate analysis)

Variable	Adjusted OR	95% CI	P-value
Age (per year increase)	1.05	1.01-1.10	0.015
Sex	1.48	0.46-4.03	0.438
Marital status	0.32	0.14-1.93	0.324
Race	0.64	0.06-6.64	0.705
Educational level	0.43	0.16-1.18	0.102
Income	0.79	0.13-4.72	0.792
Employment status	3.68	1.09-12.38	0.036
Perceived susceptibility	0.83	0.46-1.50	0.531
Perceived severity	0.81	0.32-2.02	0.649
Perceived benefits	0.87	0.36-2.09	0.753
Perceived barriers	0.41	0.18-0.93	0.032
Cues to action	4.04	1.94-8.40	0.001

P < 0.05 indicates statistical significance. OR = odds ratio; CI = confidence interval; COPD = chronic obstructive pulmonary disease; HBM = Health Belief Model.

In the adjusted multivariate analysis (Table 3), employment status (OR = 3.68; 95% CI = 1.09–12.38; P = 0.036), fewer perceived barriers (OR = 0.41; 95% CI = 0.18–0.93; P = 0.032), and stronger cues to action (OR = 4.04; 95% CI = 1.94–8.40; P = 0.001) remained significant independent predictors of influenza vaccine uptake. While increasing age was statistically associated with higher uptake (P = 0.015), the effect size was modest (OR = 1.05 per year), suggesting minimal clinical significance.

For cues to action, this domain included recommendations from healthcare providers, advice from friends or family, and willingness to receive vaccination if offered free of charge. Among these, recommendation from healthcare providers appeared to be the most influential contributor to vaccine uptake.

For perceived barriers, the domain comprised concerns that the vaccine may cause harm, cause side effects, or cause influenza. The overall effect of perceived barriers was significantly associated with lower vaccine uptake (OR = 0.41; 95% CI = 0.18–0.93; P = 0.032), with fear of side effects being the most prominent concern reported by participants.

## Discussion

In our study, 24.0% of the participants received influenza vaccination. In comparison, the US (41.7%) had the highest influenza vaccine uptake rate, followed by the UK (41.4%), while Singapore had the lowest rate (15.2%).^4,8^ In the USA, free influenza vaccination is covered by the Medicare programme.^8^ In the UK, the National Health Service is offering the influenza vaccine every year to individuals aged 50 years and above.^9^ In Malaysia, healthcare workers and individuals aged above 60 years with chronic illness are given free influenza vaccinations.

Although Singapore showed the lowest uptake rate, adult Singaporeans were able to use up to $400 of their Medisave per account for recommended vaccinations such as the influenza vaccine at all public healthcare institutions since November 2017 under the National Adult Immunisation Schedule.^10^

Diagnosing COPD in Malaysian health clinics is challenging due to limited access to spirometry. According to a 2018 report, the disease largely undiagnosed because spirometer primarily available in hospitals.^11^ A majority of COPD cases in Malaysia are managed in hospitals; fewer are treated at primary care facilities such as government clinics. The reason for the low detection rate in primary care settings could be that many people with moderate-to-severe COPD go to the hospital for follow-up care.

Our study found that employment status affected vaccine uptake, especially in high-risk fields. Other studies have shown inconclusive results, possibly due to variations in workplace vaccination policies, employer initiatives and healthcare accessibility. Certain private employers in Malaysia, particularly in high-risk industries, provide workplace vaccination programmes, which improves uptake among employees. However, vaccination rates may still differ because of how much support an employer provides and how motivated each person is. Further studies are needed to understand how different work environments and workplace vaccination programmes along with industry policies impact the number of people vaccinated.

The rate of influenza vaccination in patients with asthma and COPD in Malaysia is both similar to and different from that reported in some studies in Southeast Asia. Ang et al. discovered that employment in Singapore was associated with lower uptake of vaccination, whereas our study found otherwise.^7^ Similarly, Simmerman et al. found that cues to action by healthcare providers were a major factor for influenza vaccination in Thailand, which is consistent with our findings.^12^ A systematic review in Asia also identified fear of side effects as a key barrier to influenza vaccination.^13^ These comparisons highlight the implication that workplace-related policies, professional recommendations of healthcare providers and perceived barriers strongly influence the influenza vaccination behaviour throughout this region.^14^

Our study explored the sociodemographic factors and health beliefs associated with influenza vaccine uptake among the patients with asthma and COPD. The bivariate analysis showed that the participants who believed in the effectiveness of the influenza vaccine in preventing severe illness or complications (perceived benefits) were significantly more likely to be vaccinated (P=0.023). Likewise, those who reported fewer perceived barriers (P=0.013) and received cues to action (P=0.005) demonstrated higher vaccine uptake. The multivariate analysis further revealed that stronger cues to action (OR=4.04; 95% CI=1.94–8.40; P=0.001), fewer perceived barriers (OR=0.41; 95% CI=0.18-0.93; P=0.032) and employment (OR=3.68; 95% CI=1.09-12.38; P=0.036) were independent predictors of higher vaccine uptake. The above-mentioned findings indicate that employment status, perceived barriers, and cues to action play a significant role in influenza vaccination behaviour.

In this study, increased perceived barriers reduced influenza vaccination uptake. Fear of injection pain, concerns about side effects or the belief that the vaccine may cause illness have been reported as common barriers to influenza vaccination.^15^ This is similar to the report by Keenan, which demonstrated that a patient’s belief that influenza vaccination can make a person unwell was independently associated with not receiving the vaccine (OR=0.72; 95% CI=0.55–0.96; P=0.024).^5^ Fear of injection pain or vaccine side effects or that the vaccine will make them unwell was a barrier to vaccination.^13,14^ These misconceptions can be reduced if healthcare personnel explain the vaccine’s safety, effectiveness and availability. Clear explanations that side effects are usually mild (e.g. local pain, redness or low-grade fever) and that serious adverse effects are rare may help reduce such fear.

Cues to action, particularly recommendations from doctors, friends or family, had a strong influence on the decision to receive vaccination. While public messaging, such as posters, can serve as cues, patients may not perceive them as strongly as personal recommendations. Similarly, Lyn-Cook et al. reported that recommendation by a doctor or nurse was a significant predictor of influenza vaccination uptake.^3^ Keenan et al. also found that belief in general practitioners’ recommendations was independently associated with higher vaccine uptake. (OR=1.33; 95% CI=1.04–1.69; P=0.021).^5^ A survey conducted in South Africa by Olawale showed that 97.6% of individuals who were vaccinated were previously encouraged and advised by their doctors.^16^ Therefore, as gatekeepers to patient management, healthcare providers play an important role in promoting and recommending influenza vaccination whenever possible, especially to individuals with asthma or COPD.

Most participants agreed that they would take the vaccine if it were offered by the government free of charge. Evidence suggests that publicly funded immunization programs combined with strong public awareness are associated with higher influenza vaccination coverage.^13^ Therefore, government-subsidized annual influenza vaccination programs may help improve vaccine uptake among high-risk populations.

Our analysis showed that being employed was independently associated with higher influenza vaccine uptake. Other sociodemographic factors such as age, sex, marital status, race, educational level, income and illness did not have any significant influence on vaccination uptake. This contrasts with the report by Ang in Singapore, which revealed that being economically active was linked to lower vaccine uptake (OR=0.72; 95% CI=0.59–0.89; P=0.002).^7^ Since employment usually implies better financial stability and access, this finding may require further study.

Although Chinese and Indian involvement was lower, perhaps due to language challenges and clinic sites in mostly Malay areas, our survey revealed that Malays comprised the majority of the participants. Non-English and non-Malay speakers would have had difficulties since the study was performed in English and Malay. Furthermore, the location of the clinics might restrict the involvement of Chinese and Indian patients. These results underline the need for focused outreach including bilingual recruitment policies and clinic sites more reflecting the varied community.

### Limitations

The opportunistic sampling method employed in the study may have resulted in selection bias.

Non-random selection: Individuals were recruited on the basis of their ability and willingness to participate, rather than through chance recruitment. This means that certain groups may have been over- or under-represented. For example, patients who frequently visit clinics may have different health beliefs or experiences compared with those who do not attend clinics regularly.

Response bias: As participation was voluntary, those who provided responses were likely to have stronger views or experiences regarding influenza vaccination than those who declined to participate. This could have introduced bias, as the sample may not be representative of the entire population of patients with asthma and COPD.

Demographic limitations: The work was conducted in selected primary health care units in Klang Valley, which may have introduced bias to the results because the study population may not reflect the general population of patients with asthma and COPD in Malaysia. Patients attending these clinics may differ in socioeconomic status, health literacy or healthcare access from those in rural, urban or other geographic regions.

Self-reported data: Using self-reported data may have introduced recall bias because the participants might not accurately remember their vaccination history. Consequently, the actual vaccination rate could differ from that reported in the study.

COVID-19 pandemic: The pandemic may have influenced the patients’ willingness to participate in the study, meaning the sample may not be representative of the usual patient population. Some patients may have avoided healthcare facilities due to fear of infection, potentially reducing the diversity of the sample.

In brief, opportunistic sampling is a good way to collect data, but it has a high chance of selection bias, which can make the study less valid and less applicable. Future research should consider using stratified sampling to obtain a better overview of the population.

### Strengths

One strength of our study is the multivariate analysis. Multivariate analyses help to analyse more than two variables; the findings are closer to research predictions, and the conclusions are more accurate. Our study was able to determine the influenza vaccine uptake rate among patients with asthma or COPD residing in Klang Valley. Additionally, it explored the HBM components associated with influenza vaccine uptake in Klang Valley.

## Conclusion

Our study indicates that influenza vaccine uptake among patients with asthma or COPD is more likely when cues to action are increased; perceived barriers are fewer; and patients are employed. These findings are useful for designing future interventions to improve vaccination rates among patients with asthma or COPD, who are at a higher risk of complications from influenza infection. Using a stratified sampling design in future studies could help reduce bias. Additionally, a randomised controlled trial design could be used to study the effect of cues to action on influenza vaccine uptake.

The low rate of influenza vaccination (24%) among patients with asthma and COPD in Klang Valley invites concerns for public health. Patients are at risk for possible severe complications, hospitalisations and death. Vaccination remains a key preventive strategy to reduce influenza- related complications.

Evidence-based strategies should be implemented to develop appropriate public health policies:

- Awareness and education – Public health campaigns using local media to target vaccine hesitancy and misconceptions about vaccinations in general, especially on vaccine safety, efficacy and benefits.
- Healthcare provider engagement – Healthcare providers are important in encouraging patients to vaccinate and should be urged to proactively talk about the importance of vaccination at routine visits.
- Financial access – More emphasis should be placed on the role of vaccinators in financially subsidising access to vaccines for high-risk populations (i.e. low-income groups).
- Integration into managed care – Required vaccination should become a part of routine care in the management of asthma and COPD in primary care practice, including general practice.
- Monitoring and evaluation – Uptake of influenza vaccine and other vaccinations should be monitored to determine the impact of vaccination initiatives, and the success of interventions should be evaluated to improve clinical practice.
- Public-private engagement – Collaboration of the two sectors of healthcare for vaccine outreach should be strengthened to improve vaccine uptake.

Taking into consideration interventions of this magnitude can improve vaccination uptake among high-risk populations to alleviate the burden of managing influenza-like illnesses.

## Data sharing statement

The datasets generated and/or analyzed during the current study are not publicly available due to confidentiality and privacy considerations involving patient data but are available from the corresponding author upon reasonable request and with permission from the Ministry of Health Malaysia.

**How does this paper make a difference in general practice?**

- Healthcare professionals in primary care know the cues to action for influenza vaccine uptake, such as active doctor or nurse recommendations.
- Action can be taken to reduce barriers to influenza vaccine uptake, such as raising awareness on the benefits of vaccination and reducing fear of vaccination.
- The government can subsidise the vaccine for high-risk groups (patients with asthma and chronic obstructive pulmonary disease).
- The results can be generalised to patients in public and private settings.
- This study sets the stage for future larger population-based studies on vaccine uptake and demonstrates the collaborative approach between public and private institutions.
